# A Self-help Tool to Facilitate Implementation of eHealth Initiatives in Health Care (E-Ready): Formative Evaluation

**DOI:** 10.2196/17568

**Published:** 2022-01-17

**Authors:** Petra Dannapfel, Kristin Thomas, Alexander Chakhunashvili, Jeanette Melin, Ylva Trolle Lagerros

**Affiliations:** 1 Department of Clinical and Experimental Medicine Faculty of Medicine Linköping University Linköping Sweden; 2 Department of Health, Medicine and Caring Medical Faculty Linköping University Linköping Sweden; 3 Karolinska University Hospital Stockholm Sweden; 4 Research Institutes of Sweden Stockholm Sweden; 5 Clinical Epidemiology Division Department of Medicine Karolinska Institute Stockholm Sweden; 6 Obesity Centre Academic Specialist Centre Stockholm Health Services Stockholm Sweden

**Keywords:** implementation science, health care sector, telemedicine, organizational readiness for change

## Abstract

**Background:**

eHealth interventions have the potential to increase the efficiency and effectiveness of health care. However, research has shown that implementing eHealth in routine health care practice is difficult. Organizational readiness to change has been shown to be central to successful implementation. This paper describes the development and formative evaluation of a generic self-help tool, *E-Ready*, designed to be used by managers, project leaders, or others responsible for implementation in a broad range of health care settings.

**Objective:**

The aim of this study is to develop and evaluate a tool that could facilitate eHealth implementation in, for example, health care.

**Methods:**

A first version of the tool was generated based on implementation theory (E-Ready 1.0). A formative evaluation was undertaken through expert panels (n=15), cognitive interviews (n=17), and assessment of measurement properties on E-Ready items from 3 different workplaces (n=165) using Rasch analyses. E-Ready 1.0 was also field tested among the target population (n=29). Iterative revisions were conducted during the formative evaluation process, and E-Ready 2.0 was generated.

**Results:**

The E-Ready Tool consists of a readiness assessment survey and a hands-on manual. The survey measures perceived readiness for change (willingness and capability) at individual and collective levels: perceived conditions for change at the workplace, perceived individual conditions for change, perceived support and engagement among management, perceived readiness among colleagues, perceived consequences on status quo, and perceived workplace attitudes. The manual contains a brief introduction, instructions on how to use the tool, information on the themes of E-Ready, instructions on how to create an implementation plan, brief advice for success, and tips for further reading on implementation theory. Rasch analyses showed overall acceptable measurement properties in terms of fit validity. The subscale *Individual conditions for change* (3 items) had the lowest person reliability (0.56), whereas *Perceived consequences on status quo* (5 items) had the highest person reliability (0.87).

**Conclusions:**

E-Ready 2.0 is a new self-help tool to guide implementation targeting health care provider readiness and engagement readiness ahead of eHealth initiatives in, for example, health care settings. E-Ready can be improved further to capture additional aspects of implementation; improvements can also be made by evaluating the tool in a larger sample.

## Introduction

### Background

During the last few decades, eHealth solutions have been increasingly introduced in routine health care. Indeed, technological innovations are gradually changing the landscape of health care delivery, monitoring, efficiency, and decision-making [1,2]. eHealth has been defined by the World Health Organization as “the use of information and communication technologies for health,” and it can include a broad spectrum of different types of methods and interventions such as electronic health records, telehealth, web-based health care, and mobile health. However, research has shown that it is difficult to implement and incorporate eHealth in complex and multi-professional settings such as health care organizations [3,4].

Key barriers to implementing eHealth have been highlighted, such as perceptions of an increased workload and workflow disruptions, misalignment with clinical processes, undefined and changed roles, disruptions to face-to-face communication, and staff turnover [4,5]. However, factors that could facilitate implementation have also been identified, such as perceptions that using innovative eHealth could increase quality of care [4]. A review of reviews concluded that activities such as carefully considering the choice of an eHealth solution, engaging key stakeholders, and offering training and education, as well as allowing for adaptations of eHealth solutions, is central to succeeding with implementation efforts [6]. These recommended activities are in line with prevalent implementation theory and research arguing that factors at multiple levels in an organization together influence and contribute to implementation [7]. Furthermore, the aforementioned meta-analysis highlighted perceived benefits and harms to be especially important in eHealth implementation, for instance, perceived consequences on workflow and productivity, as well as expected costs of implementing eHealth [6].

Thus, implementation of eHealth initiatives in health care occurs at multiple organizational levels, is complex, and requires considerable work to succeed. Within implementation science, different theoretical approaches (theories, models, and frameworks) aim to describe, guide, explain, and evaluate implementation efforts [7]. These approaches can be used by implementers to plan, execute, and evaluate implementation efforts. However, in the hierarchical system of health care, clinicians and health service managers, who may have limited knowledge in implementation science, often find themselves responsible for the implementation of new digital solutions. Although, for example, a well-operationalized, multilevel framework derived from implementation theory and empirical data can guide the implementation process, lack of time, resources, and knowledge may lead to a nonsystematic implementation [8]. In addition, failures in implementation may not only lead to loss of money and time, but can also contribute to a decreased willingness among staff to adopt eHealth innovations in the future [9,10].

Furthermore, organizational readiness for change has been recognized and shown to be central to successful implementation [11-13]. It has been conceptualized by Weiner [14] as the shared determination and sense of collective capability to change. The concept of *eHealth readiness* has been proposed as important for implementing digital innovations in health care successfully [15]. A review on eHealth readiness highlighted multiple dimensions involved in the concept, such as technological aspects (eg, the extent to which technical requirements can be met), motivational aspects (eg, perceived need for technology among users), and availability of resources (eg, financial resources and competency to use technology) [15]. Tools and frameworks for measuring eHealth readiness within health care contexts also exist and are proposed to be used to facilitate implementation [15-17].

Incorporating eHealth solutions in routine health care may encompass a number of different aspects, for example, intervention conception, eHealth readiness assessment, and business and financial plans, as well as a change management plan and details on how implementation can be performed, monitored, evaluated, and sustained. Tools that assess readiness can facilitate the implementation process by guiding and informing stakeholders in a hands-on manner. In addition to improved compliance, targeted implementation efforts are more effective in terms of both costs and use of professionals’ time [18]. However, previous research has highlighted a number of challenges with regard to readiness measures, such as the measures are too theoretical, they do not address implementation issues at employee level, the scope of the measures is too broad, and the measures capture determinants for readiness rather than actual readiness [15,19].

### Objective

The aim of this study is to develop and evaluate a tool that could facilitate eHealth implementation in, for example, health care. Thus, this paper describes the development and formative evaluation of E-Ready, a generic self-help tool targeting some domains of eHealth readiness to support implementation of eHealth initiatives. The tool comprises 2 parts: (1) a survey assessing implementation readiness and (2) a hands-on manual with recommended strategies and activities to facilitate implementation.

## Methods

### Overview

The E-Ready Tool was developed to be used by managers, project leaders, and/or change leaders to facilitate and plan for implementation. E-Ready 1.0 was generated and examined in a formative evaluation process to support its validity.

### Development of E-Ready

E-Ready 1.0 was developed based on theory appraisal. This version comprised a survey assessing implementation readiness and a manual that included general information about implementation and strategies that can be used to promote practice change.

### Theory Appraisal

Theories on organizational change, implementation, and behavior change were reviewed, for instance, organizational readiness to change [14], determinants of implementation [20-23], and individual behavior change [24,25]. Theoretical constructs from these theories were listed and used to design the content and structure. For the assessment survey, items were generated to capture all the theoretical constructs that had been identified. The theoretical constructs were categorized into 5 domains: (1) capacity to change at organizational and individual levels, (2) culture to change, (3) leadership promoting change, (4) motivation to change among staff, and (5) perceived characteristics of the implementation object. These 5 domains provided an initial structure for both the survey and the manual.

### Development of the Readiness Assessment Survey

Survey items were generated for each domain and theoretical construct, that is, providing evidence based on test content [26]. For example, in the domain *implementation object,* one of the theoretical constructs was “perceived need to conduct the change among the staff.” This construct resulted in 2 proposed items: *I understand why we are implementing X at my workplace* and *I can see a need for X at my workplace.*

A total of 38 items were generated initially. Revisions were made in an iterative process and included survey structure, wording of items, wording of response options, and order of items. For example, an effort was made to tailor response options to questions. Furthermore, we revised the structure and divided the survey into main questions and subitems to facilitate the completion of the survey. The response scales were tailored to each item and were formulated to force respondents to choose an answer, that is, the items do not include a middle alternative such as “I have no opinion.” The structure of the survey no longer followed that of 5 theoretical domains; rather, the survey was constructed in a way that facilitated completion. These revisions resulted in 6 sections of questions and additional items, for instance, profession, giving a total of 33 items (Multimedia Appendix 1).

### Development of the Manual

The manual was also drafted grounded in the aforementioned 5 theoretical domains: capacity, culture, leadership, motivation, and implementation object. For example, text on why and how the factors relating to the 5 domains are important for successful implementation was included in the manual. The manual was developed with a need to know perspective so that it could also be useful for novice implementers.

### Formative Evaluation

Formative evaluation of E-Ready was undertaken in 3 ways: expert panels, cognitive interviews, and statistical tests. In addition, E-Ready was field tested among health care professionals. Revisions of the assessment survey and the manual were conducted continually during the formative evaluation process.

### Expert Panels and Cognitive Interviews

In all, 3 workshops with experts (n=15) were conducted with the aim of investigating the content validity and structure of the tool. Participants’ expertise consisted of knowledge as potential end users of the tool (physicians with clinical experience) as well as knowledge and experience in implementation. All participants had explicit responsibility for integrating eHealth in their organization. The first workshop focused on the survey items. Participants were asked to individually complete the assessment survey and then provide written feedback on how they interpreted the items and how they perceived the structure and content of the questions. The individual feedback was later discussed at the workshop. For example, to ensure that individual items captured specific theoretical constructs, the discussions focused on formulations and on how items were understood. During the second and third workshops, the manual was scrutinized. The physicians were asked to give feedback on the content of the manual, for example, the introduction of theories, information on how to use the guide, the layout, if the guide gave enough information, and if the information was clear and understandable.

Cognitive interviews were conducted with health care professionals from 2 health care settings (university hospital and specialist outpatient clinic), including physicians (n=5), registered nurses (n=4), physiotherapists (n=2), behavior therapists (n=2), and dietitians (n=4). Cognitive interviewing is a psychologically oriented method for empirically studying the ways in which individuals mentally process and respond to survey questionnaires. Cognitive interviews provide validity evidence based on response processes by allowing for potential differences in the interpretation of test items.

Data from cognitive interviewing can form the basis for appropriate modifications before further field testing [27]. Cognitive interviews were conducted by letting the participant read the question and then explaining their interpretation and meaning of the question. This technique was used in an applied sense—for the purpose of pretesting questions and determining how the items could be modified—before field testing to make the items more understandable or otherwise easier to answer. Participants were first asked to complete the survey while thinking of an eHealth initiative that they had experienced. They were then further interviewed regarding how they perceived the tool, its structure, content, and if there were survey items that were unclear or should be rephrased. Comments were noted by the researcher throughout the interview.

Iterative revisions were made in parallel with conducting expert panels and cognitive interviews. Revisions of the survey predominantly included eliminating overlap across the items and wording of the items. For example, comments based on the cognitive interviews showed that the content of 4 of the items covered similar areas, resulting in the omission of items. Revisions of the survey during this phase resulted in 29 items. Furthermore, expert data showed that the manual offered a structured way to organize the implementation process and provided insights into how to think about readiness and implementation. Revisions of the manual thus mainly covered language editing.

### Statistical Tests

To assess the measurement properties—specifically, evidence based on the internal structure [26] and fit validity [28]—of the E-Ready survey, we conducted a Rasch analysis using Winsteps software (version 4.3.1). In this analysis we included the E-Ready survey data from participants at 3 different workplaces (n=165), all of whom had filled in the E-Ready survey ahead of different eHealth implementation initiatives.

Each subscale was individually evaluated in terms of the following attributes: (1) sample to item targeting, (2) item fit to the model, (3) unidimensionality, and (4) person reliability. Each attribute is described briefly as follows:

1. The distributions of the item-threshold measures compared with those of the person measures indicate how well the items fit to the sample. The mean person measure indicates whether the sample is off-center with respect to the items. Ideally, it should be close to 0 [29].
2. Fit statistics evaluates how well the data fit the Rasch model by assessing for both inlier-pattern (INFIT)- and outlier-pattern (OUTFIT)-sensitive fit statistics. Mean-square (MNSQ) fit statistic is recommended to be between 0.5 and 1.5 to provide a productive measure. Z-standardized (ZSTD) fit statistics should be within –2 to +2 σ to support good model fit [30].
3. A principal component analysis of the fit residuals is used to assess unidimensionality. The eigenvalue is not expected to be >2 to support unidimensionality. If the eigenvalue does not support unidimensionality, high disattenuated Pearson correlation coefficient of the person measures can still prove that the dimensions are statistically the same, thus providing a unidimensional measure [31].
4. The consistency of a measure is evaluated in terms of its reliability, that is, the proportion of variance that is true variance. The reliability of the person measures should be interpreted as 0 (implies all error) or 1 (implies no error) [32].

Moreover, we hypothesized that there were positive correlations between person measures from all subscales and no differences in person measures between men and women. These analyses were conducted by means of Pearson correlation coefficients and 1-way analysis of variance in SPSS software (version 26.0; IBM Corp).

### Field Testing

As a last step, E-Ready was field tested among health care professionals. The tool was sent through email to 29 physicians and registered nurses at Karolinska University Hospital in Stockholm, Sweden. Participants were asked to complete the survey from personal experiences of implementation of an eHealth initiative. At the end of the survey, they were asked to leave comments. The feedback was positive, and no additional revisions were made. For example, positive feedback was provided regarding using the survey to engage employees in the change process. Furthermore, items investigating leadership and manager support were perceived to be specifically important.

**Figure 1:**
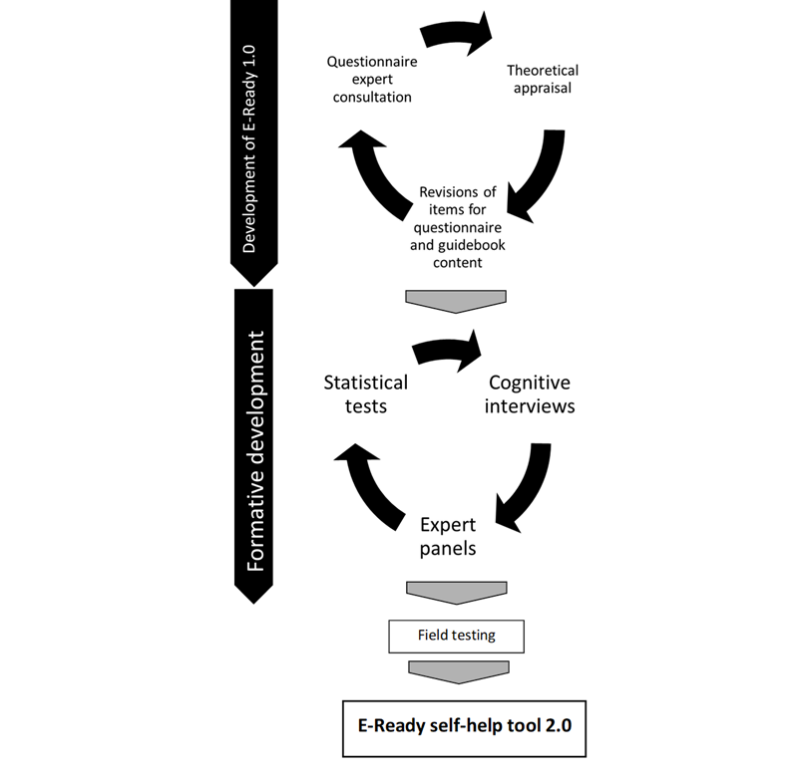
Process of the development and formative evaluation of E-Ready 1.0 to E-Ready 2.0.

Figure 1 illustrates all activities and their time points.

## Results

### Readiness Assessment Survey

The survey (Multimedia Appendix 1) aims to assess implementation readiness. The 6 sections investigate the following: (1) *Perceived conditions for change at the workplace*, for example, competency and resources for practice change (8 items); (2) *Perceived individual conditions for change*, for example, prior experience of changing practice (3 items); (3) *Perceived support and engagement among management*, for example, how urgency for change is communicated by management (5 items); and (4) *Perceived readiness among colleagues*, for example, how change is collectively valued by colleagues (5 items). There are also items aiming to capture (5) *Perceived consequences on status quo*, for example, worries regarding how practice change can influence current workflows (5 items) and (6) *Perceived workplace attitudes* toward change, for example, self-reported attitude toward the proposed change (3 items). In addition, 7 single items investigate compatibility with current work routines: commitment to change and perceived need for change, gender, years worked at current workplace, profession, and years worked in profession. In total, there are 36 items in the final assessment survey, E-Ready 2.0. It takes approximately 10 minutes to complete the survey.

**Figure 2:**
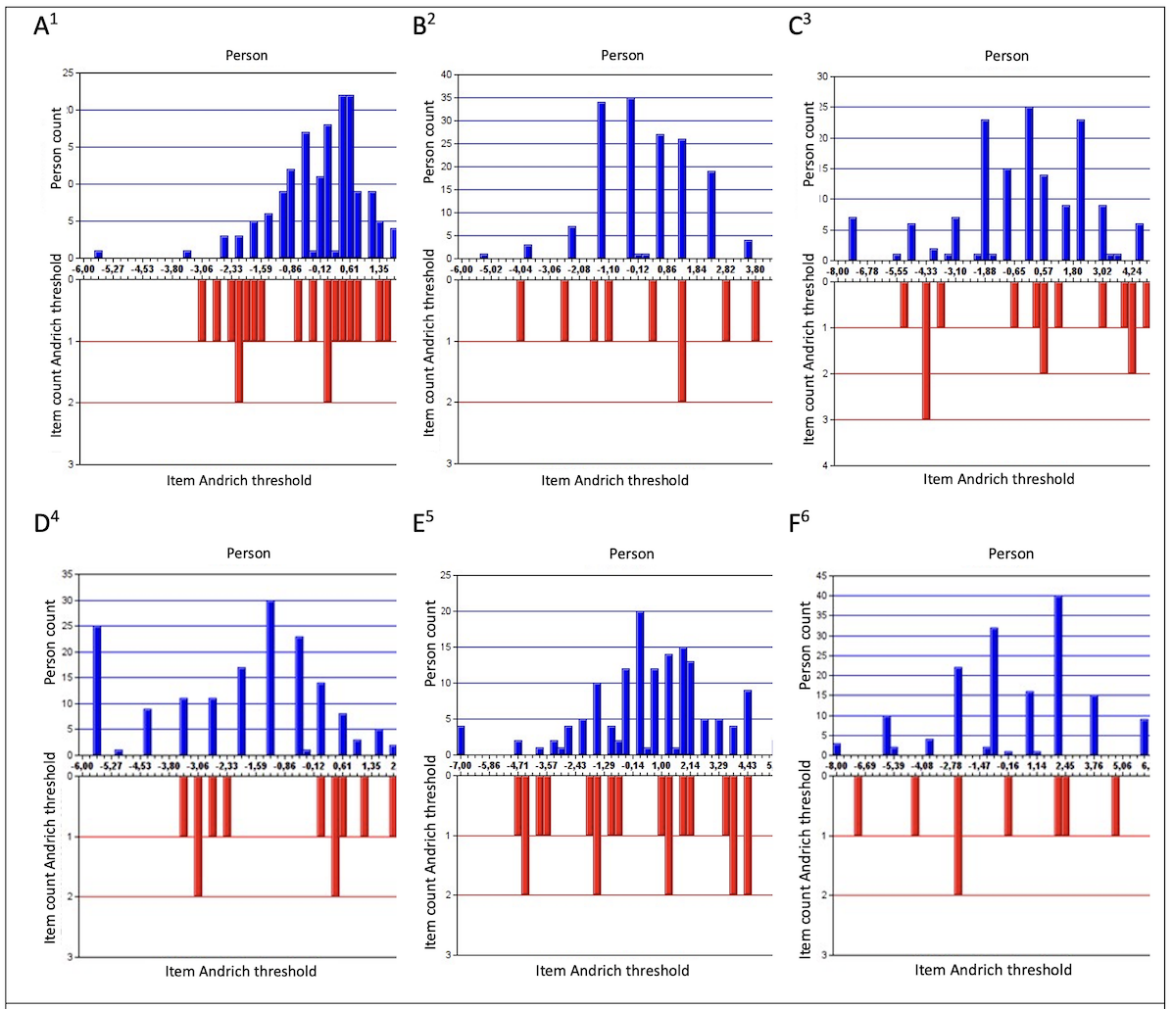
Person-item threshold histograms for each subscale. ^1^Conditions for change at the workplace; ^2^Individual conditions for change; ^3^Perceived support and engagement among management; ^4^Perceived readiness among colleagues; ^5^Perceived consequences on status quo; ^6^Workplace attitudes.

Results from the Rasch analyses showed that item-threshold measures were well covered by the person measures and person measures were fairly well covered by the item thresholds. As illustrated in the person-item threshold histograms (Figure 2A-F), there are gaps among the item thresholds. Furthermore, the subscale *Perceived readiness among colleagues* (Figure 2D) was negatively skewed (mean –1.91, SD 2.19), and *Perceived consequences on status quo* (Figure 2E) was positively skewed (mean 1.05, SD 2.88).

Table 1 provides a summary of the Rasch analysis of the subscales. In short, the subscale Conditions for change at the workplace had all INFIT and OUTFIT MNSQ values within the acceptable range, and the 2 items with INFIT and OUTFIT ZSTD values were slightly outside 2 σ. By a closer inspection of the principal component analysis loadings, we found that a cluster of 3 items addressed the conditions among the employees, whereas the others addressed organizational conditions. The subscale Individual conditions for change also had all INFIT and OUTFIT MNSQ values within the acceptable range, but 2 items with INFIT and OUTFIT ZSTD values were slightly outside 2 σ. The person reliability was lowest for this scale, which, speaking reasonably, is a consequence of having only 3 items. The subscale Perceived support and engagement among management showed some statistical item misfit but supported unidimensionality. By removing the most misfitting item (item E of the E-Ready survey, which addresses management competence and knowledge unlike the others that address management actions), we found that the fit statistics were improved, and the person reliability increased from 0.84 to 0.86.

**Table 1:** Summary of the Rasch analysis of the E-Ready subscales.

			Conditions for change at the workplace, 8 items		Individual conditions for change, 3 items		Perceived support and engagement among management, 5 items		Readiness among colleagues, 5 items		Perceived consequences on status quo, 5 items		Workplace attitudes, 3 items
**Item fit statistics**													
	MNSQ^a^ INFIT^b^, mean (SD)	1.00 (0.17)		1.00 (0.26)		0.99 (1.22)		0.99 (0.27)		0.99 (0.42)		0.98 (0.04)	
	ZSTD^c^ INFIT, mean (SD)	–0.10 (1.60)		–0.20 (2.30)		–0.20 (1.40)		–0.20 (2.10)		–0.60 (3.60)		–0.20 (0.40)	
	MNSQ OUTFIT^d^, mean (SD)	0.99 (0.18)		1.02 (0.29)		1.00 (1.25)		0.98 (0.27)		1.02 (0.46)		1.03 (0.09)	
	ZSTD OUTFIT, mean (SD)	–0.10 (1.70)		0.00 (2.50)		–0.2 (1.40)		–0.40 (2.10)		–0.40 (3.80)		0.20 (0.50)	
	MNSQ INFIT (number of items misfitting)	0		0		1		0		1		0	
	ZSTD INFIT (number of items misfitting)	2		2		2		3		3		0	
	MNSQ OUTFIT (number of items misfitting)	0		0		1		0		1		0	
	ZSTD OUTFIT (number of items misfitting)	2		2		2		3		4		0	
**Unidimensionality**													
	Eigenvalue unexplained variance in first contrast	2.43		1.78		1.65		2.37		2.22		1.48	
	Disattenuated correlation cluster, 1-3	0.21		0.13		0.66		0.37		0.61		1.00	
**Person reliability**													
	Reliability index	0.69		0.56		0.84		0.72		0.87		0.77	
	Separation index	1.50		1.13		2.30		1.60		2.55		1.85	

^a^MNSQ: mean-square.

^b^INFIT: inlier-pattern-sensitive fit.

^c^ZSTD: Z-standardized.

^d^OUTFIT: outlier-pattern-sensitive fit.

The subscale *Readiness among colleagues* had acceptable INFIT and OUTFIT MNSQ values but INFIT and OUTFIT ZSTD misfit and suffered from unidimensionality. The items comprised both specific tasks (eg, having discussions among colleagues) and overall collaboration, which might explain the weaknesses. The subscale *Perceived consequences on status quo* showed the highest person reliability, but, at the same time, it suffered from several item misfittings. This could possibly be explained by the nature of the response options and statements, that is, the extent to which the implementation of X would affect several working tasks might not be quantified in a common scale for different implementations at different workplaces. Finally, the subscale *Workplace attitudes* did not have any misfitting items; it supported unidimensionality and had a person reliability close to the desired 0.8.

As shown in Table 2, correlations among the person measures were low to moderate for all subscales except *Perceived consequences on status quo*. This subscale showed some negative correlations, especially toward *Workplace attitudes* (*r*=–0.30). Statistical differences between the person measures of men and women were present for 1 subscale, *Individual conditions for change*, where the men had higher measures than the women.

**Table 2:** Correlations among person measures for the E-Ready subscales.

	Conditions for change at the workplace	Individual conditions for change	Perceived support and engagement among management	Readiness among colleagues	Perceived consequences on status quo	Workplace attitudes
Conditions for change at the workplace	1.00	—^a^	—	—	—	—
Individual conditions for change	0.48	1.00	—	—	—	—
Perceived support and engagement among management	0.50	0.28	1.00	—	—	—
Readiness among colleagues	0.29	0.21	0.53	1.00	—	—
Perceived consequences on status quo	–0.16	–0.02	0.01	–0.02	1.00	—
Workplace attitudes	0.52	0.31	0.41	0.27	–0.30	1.00

^a^Not applicable.

### The Manual

The manual is in the form of a 56-slide PowerPoint (Microsoft Corp) document. The manual predominantly includes recommendations for strategies and activities that facilitate implementation. Recommendations are tailored to low-score areas as identified by the survey. The manual includes the following:

- A brief introduction, including aspects of organizational readiness to change and implementing innovations in practice. This section is written as executive summaries, for example, “Don’t focus solely on technology” and “Involve staff from the beginning.”
- Step-by-step instructions on how to use the E-Ready Tool. For example, what to do, when, and how, as well as suggestions on how to communicate the E-Ready scores to personnel.
- Text that describes the themes of the E-Ready Tool and why they are essential when planning a change process: (1) capacity to change (organizational and individual), (2) culture to change, (3) leadership promoting change, (4) motivation to change, and (5) characteristics of the implementation object.
- Guidance on how to create an implementation plan, including linking your plan to your E-Ready scores and the E-Ready themes.
- A narrative of a case example using the E-Ready Tool, including how readiness assessment can guide and inform an implementation plan.
- “12 Tips for Success,” for example, how to engage staff or measure practice change.
- Recommended reading for those who wish to learn more about implementation theory and a more extensive explanation of the theories underpinning the tool. This part includes scientific references.

### How to Use the E-Ready Tool

Using the E-Ready Tool involves 6 steps where the person responsible for the implementation performs the following actions:

1. Informs relevant personnel in the organization about the plan to use the E-Ready Tool in conjunction with the upcoming eHealth implementation.
2. Reads the manual to gain basic knowledge of readiness and implementation and obtain instructions on how to use E-Ready.
3. Informs the personnel about the upcoming eHealth implementation and the use of the E-Ready Tool in the implementation process. The assessment survey is then sent to the personnel.
4. Receives a summary report of the results in the form of a report that visualizes the results. The results indicate which areas for implementation the personnel have estimated as high or low in terms of readiness.
5. Analyzes the results with the implementation team (if there is one), uses the guide, and discusses which activities can be performed to improve readiness or implementation.
6. Creates an implementation plan with the use of the guide. The guide also includes instructions on how to follow through and measure the implementation process.

## Discussion

### Description of Development and Formative Evaluation of E-Ready

Here, we describe the development and formative evaluation of a novel implementation tool: E-Ready. The tool is a theory-based self-help tool to measure individual and organizational readiness to facilitate eHealth implementation in, for example, health care. The tool consists of a readiness assessment survey and a hands-on manual. The survey measures perceived readiness for change (perceived willingness and capability) in a workplace setting at individual as well as collective levels.

eHealth readiness assessment can be seen as a holistic approach from intervention conception to evaluation and monitoring, as well as sustainability of implementation. The E-Ready Tool considers some key aspects of eHealth readiness, such as perceived conditions for change at group and individual levels, support and engagement level among management, and perceptions among stakeholders about the change per se. In addition, the manual can be used to guide the generation of implementation and evaluation plans. Thus, the tool primarily considers implementation in the local health care setting using a stakeholder perspective. That is, the tool does not explicitly consider other dimensions of eHealth readiness that have been highlighted in previous research, such as governance or societal readiness [33].

The E-Ready Tool was developed from a need to facilitate systematic implementation of eHealth in health care and the urge to meet health care challenges with regard to new digital solutions. Previous research has highlighted difficulties and delays in integrating new technology with existing workflows, tasks, and organizational processes [3,4,34]. Furthermore, studies suggest that measuring and considering the readiness for change within an organization or workplace can facilitate implementation [12,13]. Readiness assessments can thus help to predict and plan for implementation [15]. However, future studies will need to assess the effect of measuring tools such as E-Ready on implementation outcomes, for example, the reach, adoption, and acceptability of eHealth interventions among health care professionals and patients [35]. For example, more knowledge is needed on how different dimensions of organizational readiness (eg, capacity or willingness among staff) influence implementation outcomes in general and at different levels of an organization over time and at specific time points, as well as how the degrees of readiness differ within an organization and how this variance influences implementation outcomes and potentially can be addressed. In addition, an important aspect to be considered for future research is at what time point readiness assessment is the most valuable. As any measurement only captures readiness for the next step of a change process rather than readiness for the implementation as a whole, multiple measurements of readiness may be needed. E-Ready and similar instruments could be used to investigate these knowledge gaps to further our knowledge on the relationship between organizational readiness for change and implementation.

Previous research has highlighted several challenges with existing readiness-measuring tools, for instance, the importance of tools with regard to targeting and capturing issues at employee level, acknowledging that readiness is change- and situation-specific, and the need for tools to capture readiness (eg, perceptions about capability for change) rather than its determinants (eg, resources for enabling change) [15,19]. We have attempted to address these issues in the development of the E-Ready Tool. Our ambition is to provide a brief, theory-based tool that can be used by stakeholders who are novices at implementation. Specifically, E-Ready 2.0 combines readiness assessment with a hands-on manual on how to plan and promote implementation. Combining these 2 components could offer stakeholders accessible and concrete guidance on implementation. The themes that are assessed in the survey and discussed in the manual, although theory-based, were perceived to be relevant among end users, suggesting that E-Ready addresses appropriate employee-level implementation issues. Furthermore, although the themes of the questions in the survey are generic and can be applied to a broad range of eHealth initiatives, the eHealth initiative in question is to be specified at the top of the assessment survey, which will tailor the questions and guide responders toward specific eHealth initiatives. We have striven to enable E-Ready to measure and address readiness for change rather than its determinants mainly by reviewing relevant theory [14] and linking theoretical constructs to the survey items and manual content. Finally, the tool is generic and could potentially also be used as a change management component of implementation in fields other than health care, although the testing so far has been conducted in the health sector. Future studies will have to further investigate the validity and reliability of the E-Ready Tool in larger effectiveness studies.

By considering the fairly well-targeted sample to item and by following the general principle of Rasch fit statistics, we found acceptable measurement properties of E-Ready in terms of internal structure and fit validity. However, both the assessment of unidimensionality and provision of a highly reliable measure suffer from including few items in some of the subscales. Thus, there is room to further extend the survey with additional items to improve the measurement properties of the E-Ready subscales. There is indication of construct underrepresentation [28]; thus, items investigating perceptions of privacy issues associated with eHealth interventions, for example, could be added to improve content coverage and content validity. Moreover, despite the practical significance of measures of different aspects with the subscales, the moderate correlations among person measures indicate that a couple of items could be combined to provide a higher-ordered E-Ready measure. This can reduce the measurement uncertainties and further improve the reliability; however, at the same time, a higher-ordered E-Ready measure might not be of the same clinical significance as measures of subscale attributes.

### Methodological Considerations

Among the limitations is the relatively small number of respondents involved in the initial formative evaluation. Considering the qualitative methodology used in the evaluation (ie, expert panels and cognitive interviews), we estimated that the number of participants included would be sufficient to achieve our aim. However, a larger number of participants could have improved generalizability; especially if participants from various health care settings and professions had been included, the rigor would have been enhanced. Thus, continued development work to, for example, better assess the validity and reliability of the survey is needed, as is research on the usability of the manual.

Potential strengths of the study are that end users were included in the formative evaluation, in the expert panels as well as in the cognitive interviews. Involving end users could have increased the usability and acceptability of the tool. However, this needs further investigation in future studies. Using qualitative data from both clinicians and implementers improved and gave support that the content and structure of the tool were relevant and understandable for end users.

Another strength was that we applied the Rasch analysis as part of development. This is not simply a mathematical or statistical approach; rather, it is a specifically metrological approach to human-based measurement [36]. The Rasch analysis provides separate measures of persons and items scaled on the conjoint interval logit scale [37], which in turn provides objective measures that can be used for arithmetic operations.

The Rasch analysis can be viewed as a statistical model used for validating assessment tools [38]. In this work we have addressed some aspects of validity, that is, the expert group provided *evidence based on test content*, the cognitive interviews provided *evidence based on response processes*, and the Rasch analyses provided *evidence based on internal structure* [26,39]. However, we have not evaluated the 2 remaining validity aspects in the *Standards for Educational and Psychological Testing* [26], namely relations to other variables or consequences of testing. Furthermore, the evidence provided on validity aspects in this study may benefit re-evaluations with added items and in larger samples. Moreover, it should also be noted that validity evaluations of relations to other variables is not a straightforward process because the constructs purported to be measured with E-Ready do not have a gold standard to be compared with. We therefore encourage further work with a focus on construct modeling [40] and qualitative understanding of implementation readiness theory.

### Conclusions

E-Ready 2.0 is a new self-help tool to guide implementation targeting health care provider readiness and engagement readiness ahead of eHealth initiatives in, for example, health care settings. E-Ready could be improved further to capture additional aspects of implementation; improvements could also be made by evaluating the tool in a larger sample.

## Appendix

Multimedia Appendix 1 E-Ready 2.0: Readiness assessment survey.

## Abbreviations

INFIT: inlier-pattern-sensitive fit
MNSQ: mean-square
OUTFIT: outlier-pattern-sensitive fit
ZSTD: Z-standardized
