# Supplementary material for: A Self-help Tool to Facilitate Implementation of eHealth Initiatives in Health Care (E-Ready): Formative Evaluation
Source: JMIR Form Res. 2022 Jan 17;6(1):e17568. doi: 10.2196/17568 (PMC8804954; doi:10.2196/17568)
Supplement: Multimedia Appendix 1 [file formative_v6i1e17568_app1.docx]

**E-Ready^©^**^[[1]](#footnote-1)^ **Questionnaire**

| 1. How do you perceive the **resources in your workplace** to implement X? | | | | |
| --- | --- | --- | --- | --- |
|  | Does not exist at all | Exist to a small extent | Exist to a fairly large extent | Exist to a large extent |
| a) Sufficient allocated time to implement X | □ | □ | □ | □ |
| b) Sufficient staff resources to implement X | □ | □ | □ | □ |
| c) Clear goals how to implement X | □ | □ | □ | □ |
| d) Experience among colleges to work with digital solutions | □ | □ | □ | □ |
| e) Experience among colleges to implement new work routines | □ | □ | □ | □ |
| f) Adequate competency to implement X | □ | □ | □ | □ |
| g) Sufficient support and guidance to adapt X to our workplace | □ | □ | □ | □ |
| h) Sufficient training to be able to use and work according to X | □ | □ | □ | □ |
| Comments: ________________________________________________________________________________________________________________________________________________________________________________ | | | | |

| 2. How do you perceive **your own resources** to use X in your work? | | | | |
| --- | --- | --- | --- | --- |
|  | Does not exist at all | Exist to a small extent | Exist to a fairly large extent | Exist to a large extent |
| a) Experience to work with digital solutions | □ | □ | □ | □ |
| b) Competency to work with X | □ | □ | □ | □ |
| c) Feel comfortable to work with X | □ | □ | □ | □ |
| Comments: ________________________________________________________________________________________________ | | | | |

| 3.To what extent do you perceive your **manager** … | | | | |
| --- | --- | --- | --- | --- |
|  | Not at all | To some extent | Fairly large extent | Large extent |
| a) Communicates the need for implementing X | □ | □ | □ | □ |
| b) Encourages staff to engage in activities to implement X | □ | □ | □ | □ |
| c) Takes an active role in implementing X | □ | □ | □ | □ |
| d) Clearly communicates with staff how X will be implemented | □ | □ | □ | □ |
| e) Have insights in how X will influence status quo | □ | □ | □ | □ |
| Comments: ________________________________________________________________________________________________ | | | | |
|  | | | | |

| 4. To what extent do you perceive your **colleagues**… | | | | |
| --- | --- | --- | --- | --- |
|  | Not at all | To some extent | Fairly large extent | Large extent |
| a) Discuss how work routines need to change when implementing X | □ | □ | □ | □ |
| b) Discuss about duties that need to be omitted when implementing X | □ | □ | □ | □ |
| c) Discuss about new duties that needs to be done when implementing X | □ | □ | □ | □ |
| d) Work together to adapt current work routines to X | □ | □ | □ | □ |
| e) Take a collective responsibility for the implementation | □ | □ | □ | □ |
| Comments: ________________________________________________________________________________________________ | | | | |

| 5. In what way do you think implementing X will **impact your daily routines** regarding... | | | | | | | | | |
| --- | --- | --- | --- | --- | --- | --- | --- | --- | --- |
|  | Considerably worse | | Somewhat worse | | Unchanged | | Slightly better | | Much better |
| a) Your ability to work in accordance with your values | □ | | □ | | □ | | □ | | □ |
| b) Your ability to exercise your professional role | □ | | □ | | □ | | □ | | □ |
| c) Your ability to offer high quality care | □ | | □ | | □ | | □ | | □ |
| d) Your ability to manage all your work | □ | | □ | | □ | | □ | | □ |
| Comments: ________________________________________________________________________________________________ | | | | | | | | | |
| 6. How do you perceive attitudes to implement X at **your workplace?** | | | | | | | | | |
|  | | Very  negative | | Rather negative | | Rather positive | | Highly positive | |
| a) Among colleagues | | □ | | □ | | □ | | □ | |
| b) Your attitude | | □ | | □ | | □ | | □ | |
| c) Your manager’s attitude | | □ | | □ | | □ | | □ | |
| Comments: ________________________________________________________________________________________________ | | | | | | | | | |

|  | Not at all | Quite bad | Quite good | Very good |
| --- | --- | --- | --- | --- |
| 7. How well does X fit with current work routines? | □ | □ | □ | □ |
| Comments: ________________________________________________________________________________________________ | | | | |

|  | Passive | Rather passive | Rather active | Active |
| --- | --- | --- | --- | --- |
| 8. What role would you like to have when X is being implemented at your workplace? | □ | □ | □ | □ |
| Comments: ________________________________________________________________________________________________ | | | | |

|  | Pointless | Some value | Fairly great value | Great value |
| --- | --- | --- | --- | --- |
| 9. How do you perceive the value of implementing X at your workplace? | □ | □ | □ | □ |
| Comments: ________________________________________________________________________________________________ | | | | |

|  | Female Male Other Do not know |
| --- | --- |
| 10. I am | □ □ □ □ |
| 11. Years working at my current workplace | ________________________________ |
| 12. Profession | ________________________________ |
| 13. Years in practice | ________________________________ |

1. Although copyrighted, E-Ready may be freely used if appropriate credit is given. Please contact Dr. Petra Dannapfel (petra.dannapfel@sll.se) if you intend to use E-Ready. Please note that the English translation presented here is for the purpose of this paper only. Translations of E-Ready from Swedish to several languages is currently under way. [↑](#footnote-ref-1)
